# A lexicon obtained and validated by a data-driven approach for organic residues valorization in emerging and developing countries

**DOI:** 10.3389/frai.2025.1557137

**Published:** 2025-09-02

**Authors:** Christiane Rakotomalala, Jean-Marie Paillat, Frédéric Feder, Angel Avadí, Laurent Thuriès, Marie-Liesse Vermeire, Jean-Michel Médoc, Tom Wassenaar, Caroline Hottelart, Lilou Kieffer, Elisa Ndjie, Mathieu Picart, Jorel Tchamgoue, Alvin Tulle, Laurine Valade, Annie Boyer, Marie-Christine Duchamp, Mathieu Roche

**Affiliations:** ^1^Centre de coopération Internationale en Recherche Agronomique pour le Développement (CIRAD), Unité Propre de Recherche (UPR) Recyclage et Risque, Saint-Denis, La Réunion, France; ^2^Recyclage et Risque, Centre de coopération Internationale en Recherche Agronomique pour le Développement (CIRAD), Université de Montpellier, Montpellier, France; ^3^Centre de coopération Internationale en Recherche Agronomique pour le Développement (CIRAD), Unité Propre de Recherche (UPR) Recyclage et Risque, Montpellier, France; ^4^Centre de coopération Internationale en Recherche Agronomique pour le Développement (CIRAD), Unité Propre de Recherche (UPR) Recyclage et Risque, Angers, France; ^5^ISTOM, École Supérieure d'Agrodeveloppement International, Angers, France; ^6^Institute of Agrifood Research and Technology (IRTA), Sustainability in Biosystems Research Program, Torre Marimon, Caldes de Montbui, Barcelona, Spain; ^7^Centre de coopération Internationale en Recherche Agronomique pour le Développement (CIRAD), Unité Propre de Recherche (UPR) Recyclage et Risque, Dakar, Sénégal; ^8^Centre de coopération Internationale en Recherche Agronomique pour le Développement (CIRAD), Direction Générale Déléguée Recherche et Stratégie (DGDRS), Délégation á l'Information scientifique et á la SCience Ouverte (DiscO), Montpellier, France; ^9^Centre de coopération Internationale en Recherche Agronomique pour le Développement (CIRAD), Unité Mixte de Recherche (UMR) Territoires, Environnement, Télédétection et Information Spatiale (TETIS), F-34398 Montpellier, France; ^10^Territoires, Environnement, Télédétection et Information Spatiale (TETIS), Université de Montpellier, AgroParisTech, CIRAD, Institut National de Recherche pour l'Agriculture, l'Alimentation et l'Environnement (INRAE), Montpellier, France

**Keywords:** text mining, organic waste, biological transformation, agriculture, valorization

## 1 Introduction

Open dump remains the main management process of organic residue in middle- and low-income countries (Kaza et al., 2018). Indeed, according to this study, municipal solid waste is composed of 44% organic fraction. However, waste recycling or valorization is about 7, 4.7, and 21% in Sub-Saharan Africa, Caribbean/Latin America, and South Asia respectively. It is thus interesting to determine organic residue valorization status in those regions. Answer to that question could be prospected through textual analysis. The method herein represents the first step to that end. Indeed, when missing, text mining could be used to extract thematic lexicon from a bibliographic corpus to drive a state-of-art in the valorization of organic residues in agriculture in developing countries. In this work, text mining and Natural Language Processing (NLP) methods enable to generate a specialized lexicon on this specific area. The definition of relevance of terms is challenging and discussed in this data paper. Actually, terminology extraction methods are generally based on benchmarks (i.e., gold-standard) or terms manually validated (Nazar and Lindemann, 2022) but an experimental protocol that takes into account different kinds of relevance to consolidate the process is understudied. This needs to integrate expertise knowledge, agreement of experts regarding definitions and evaluation associated with, and the task to do. This paper highlights how this construction is conducted by considering different point-of-view of relevance in a multidisciplinary context. It is important to notice that this kind of lexicon specifically focused on organic residues valorization does not exist in agriculture semantic resources like AgroPortal which include more than 200 ontologies/thesaurii/lexicons (Jonquet et al., 2018).

The present work consisted of using text mining approach to construct thematical lexicon from a corpus related to valorization of organic waste in developing countries. Method used to collect data was detailed first, followed by a section about the technic adopted to select, annotate, and validate the lexicon. Finally, future perspective work was explained in a concluding section. This exploratory methodology could be used to guide a more in-depth and oriented text analysis of scientific publications (i.e., scientometric analysis). Moreover, this methodology can be reused and/or adapted in other domain depending on purpose. In our ongoing work, we use this lexicon to conduct a semantic analysis of scientific publications dealing with organic residues valorization in emerging and developing countries.

## 2 Proposed method to collect the data

### 2.1 Construction of the corpus

Several online databases were consulted in 2021, to extract articles relating to biotransformation and valorization in agriculture of organic residues in emerging and developing countries (WoS, Ovid, Scopus, Google scholar, HAL, Cairn.info, AGRIS, and Agritrop^1^) published until 2021. Terms used for bibliographic search in all databases through specific queries are detailed in the Appendix section of this paper.

The equation used in the Web of Science collection was thereafter adapted for the other databases specificities. Advanced search was not available for most of the free online database, a global thematical search was then adopted (Appendix 1). The search gave 24,186 references on which a selective sorting was conducted to avoid duplicates and to select references in English only. A total of 7,692 references were used to generate the dataset available in the excel file (Initial_Corpus_References.xlsx) available on depository (Rakotomalala et al., 2023). The corpus of the dataset combines articles, reports, book sections, and student thesis with bibliographic references (authors, year of publication, title, doi, and url).

### 2.2 Extraction of candidate terms

BioTex (Lossio-Ventura et al., 2014) was used to perform an Automatic Term Extraction (ATE) on the corpus. The terms extracted (e.g., rumen, humic acid, nutrient recovery, …) give a semantic point of view of the theme of the text. This tool was developed for Biomedical term extraction (Lossio-Ventura et al., 2016) and was adapted to extract terms associated with food security (Roche et al., 2022). First, BioTex performed a linguistic screening through syntactic patterns (noun-noun, adjective-noun, …). In order to rank terms extracted on the “titles” corpus, the F-TF-IDF-C score integrated to BioTex was applied. This measure combines (i) C-value (4) to favor multi-word terms extracted, and (ii) TF-IDF (Term Frequency-Inverse Document Frequency) to highlight discriminative terms (Lossio-Ventura et al., 2016).

Text mining was thereafter performed on titles of the corpus using the BioTex tools (Lossio-Ventura et al., 2014) and the result can be found in the associated excel file (Extracted_Terms.xlsx) on depository (1). The first column contains the 19,580 terms obtained from the extraction. The second column (“term”) presents the terms constituted of words or compound nouns (e.g., mulch, effluents, soil amendments, bagasse co-composting). The rank, in the last column, is obtained by maximizing a discriminative score associated with terms (i.e., F-TF-IDF-C).

## 3 Terminology selection and analysis

### 3.1 Annotation guide V1 and associated Fleiss Kappa

Five specialist raters conducted a first annotation on 200 sampled candidate terms among the 19,580 to exclude irrelevant terms to the topic of interest following the guideline file (Annotation_guidelines.pdf) available on the depository (Rakotomalala et al., 2023). Specialists were researchers in “Recyclage et risque” unit of Cirad in France, working on recycling organic residue in agriculture and associated risks. The group was specialized in biochemistry, agronomy, microbiology, ecologist, soil science, and environmental assessment using both monitoring and modeling approaches. Each rater was asked to categorize each candidate term belonging to i) organic residues (OWT) and/or ii) biotransformation process (TM) and/or iii) valorization in agriculture (AV) or iv) none of them (None) following the first annotation guide. Definition of each category is described in the annotation guidelines. Table 1 shows example of the first step of annotation conducted by specialist.

**Table 1:** Examples of annotation process.

	**Expert 1**			**Expert 2**			**Expert 3**	**Expert 4**		**Expert 5**	
**Term**	**Category 1**	**Category 2**	**Category 3**	**Category 1**	**Category 2**	**Category 3**	**Category 1**	**Category 1**	**Category 2**	**Category 1**	**Category 2**
Manure	OWT	TM	AV	OWT			OWT	OWT	AV	OWT	
Anaerobic digestion	TM			TM			TM	TM		TM	
Biogas	TM			TM			None	None		TM	
Rice	AV			OWT	AV		OWT	None		AV	OWT
Nitrogen	OWT	TM	AV	OWT	TM	AV	None	None		AV	TM

Category designated if the terms belong to Organic waste type (OWT), Transformation method (TM), Agricultural valorization (AV), or none of them.

The Fleiss Kappa (Fleiss, 1971) which measures agreement between several raters equals to 0.52 for this first annotation corresponding to a bad agreement between the 5 raters. The 4 categories chosen to annotate the candidate terms appeared to be too restrictive. Terms indirectly associated to one or more of the 4 categories have been excluded by several raters.

### 3.2 Annotation guide V2 and associated Fleiss Kappa

In a second annotation guideline, the manual labeling process focuses on the overall degree of pertinence related to the topic of valorization of organic residues. In this context, candidate term was annotated according 3 classes: (i) very pertinent when it was directly connected to one or more category(ries) (i.e., OWT+, TM+, AV+), (ii) pertinent when it was indirectly connected to one or more category(ries) (i.e., OWT, TM, AV), and (iii) irrelevant (i.e., None).

A second annotation on the same 200 sampled terms was conducted. All results of the two series of annotation can be viewed with the file “Raters_Annotation_Results.xlsx” in our dataset (Rakotomalala et al., 2023). Fleiss Kappa was calculated for 3 and 5 raters. It revealed a decreasing trend of the value (0.84 to 0.60) with increasing number of raters. Closer comparison highlighted more terms indirectly related to one or more category(ies) selected by 3 raters with high value of Kappa. In order to include as many terms indirectly related to the subject as possible, it was decided to apply the logic of these 3 annotators to pursue the categorization of the remaining terms.

In Table 2, the results are evaluated in terms of precision (percentage of pertinent terms) obtained over the top k extracted terms (P@k). The results confirm that the ranking function of BioTex is adapted by highlighting relevant terms at the top of the list. For instance, precision value with *k* = 100 and *k* = 200 is high (more than 80%) but recall will be low because a lot of relevant terms are not proposed. Actually, a precise recall value is difficult to calculate because we do not have gold standard.

**Table 2:** Precision (P@k) according the BioTex ranking.

**Rank of term—k**	**Precision—P@k (%)**
100	83
200	81
500	59
1,000	49
3,000	41
5,000	36
10,000	20
19,580	25

The above detailed dataset can be found in the CIRAD Dataverse repository (Rakotomalala et al., 2023).

### 3.3 Annotation and validation of all extracted terms

One of the five specialists then pursued the annotation, with a degree of relevance, on the remaining extracted terms. It was decided to continue the categorization with the degree of pertinence and to apply the logic of the three annotators with the high kappa value explained above. It took about 1-week work for the rater to conduct the categorization. The same five raters were then asked to verify and finalize the terms selection related to the biotransformation and valorization in agriculture of organic residues in low-income countries. All verified relevant terms were combined in the last file on the depository (Pertinent_Terms.xlsx), containing terms which can be indirectly (first sheet) or directly (second to fourth sheet) related to the topic.

From the 19,580 initial candidate terms, about 75% were not associated to the topic of interest (Table 2). Irrelevant terms included words which are not related to organic residues nor biotransformation nor valorization in agriculture, such as: absence, certification, design, effect, fecundity, fitness, gray, immune response, integration analysis, low cost, marker genes, …. Among the 25% relevant terms, 2,079 were closely associated with the organic residues valorization in emerging and developing countries such as sludge, sewage, livestock, manure, slurry, anaerobic digestion, composting, vermicomposting. Several terms can be found in the glossary of terms related to livestock and manure management (Pain and Menzi, 2011) and figure among terms with high pertinence in this dataset. Moreover, some of relevant terms are cited in literatures as the biotransformation (e.g.,: anaerobic digestion, composting, bioethanol, biohydrogen) and valorization in agriculture (e.g.,: biofertilization, organic fertilizers, amendments) of organic residues (e.g., rice straw, sugarcane bagasse, animal manure; Chew et al., 2019; Chavan et al., 2022).

**Figure 1:**
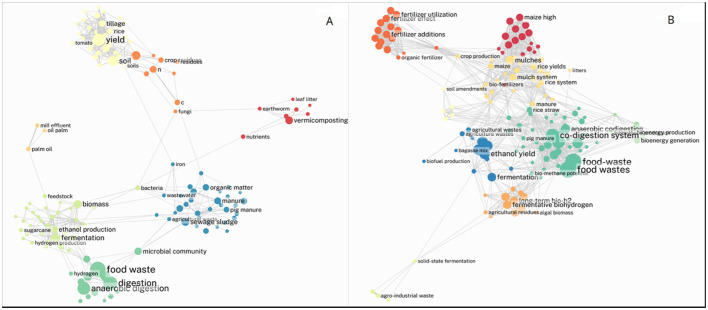
Examples of network mapping from CorTexT driven by the lexicon obtained with this study **(A)** and with only very pertinent terms of the lexicon **(B)**.

The produced lexicon is currently used in a semantic-driven analysis of our corpus based on the CorTexT software (Breucker et al., 2016). In the context of this multidisciplinary work on-going, we obtain deeper knowledge regarding bioconversion and valorization in agriculture of organic residues in low-income countries as highlighted in Figures 1A, B.

## 4 Conclusion and future work

The text-mining tool used in this work is based on statistical criteria that highlight discriminative terms. This method identifies significant terms that are present in the texts. As future work, the proposed framework could be extended by extracting variation of terms (León-Araúz et al., 2020) that enables to recognize rare and/or unsystematic terms but also synonyms. Moreover, embedding approaches (Sarkar et al., 2019), language models (Devlin et al., 2019), generative methods based on LLM (Large Language Models) techniques (Giguere and Iankovskaia, 2023) could be applied to recognize new terms. Language model techniques are based on generic models like BERT—Bidirectional Encoder Representations from Transformers (Devlin et al., 2019) or specific ones like AgriBERT—Knowledge-Infused Agricultural Language Models for Matching Food and Nutrition (Rezayi et al., 2022) dedicated to the agriculture domain. These models can be fine-tuned for specific tasks like terminology extraction. There can be used to improve terminology extraction. Note that the use of language models could be relevant for specific NLP tasks and domains like the agriculture area (De et al., 2025). As future work, we plan to compare the applied methods described in this paper with other approaches based on language models but also Large Language Models (LLM) for terminology extraction (Tran et al., 2025). LLM could also be used to expand our initial lexicon. This enables to extract variations of exiting terms and synonyms but also new terms. In the context of our work, the objective is to conduct a semantic analysis of terms present in the corpus, so the use of words or phrases in our lexicon but not used in our dataset (i.e., corpus) is not really useful.

## Data Availability

The datasets presented in this study can be found in online repositories. The names of the repository/repositories and accession number(s) can be found at: https://doi.org/10.18167/DVN1/HNZZSI.

## Footnotes

^1^http://webofknowledge.com/WOS, https://ovidsp.ovid.com/autologin, https://scholar.google.com/, https://hal.science/, https://www.cairn.info/, https://agris.fao.org/, http://agritrop.cirad.fr/
