# Supplementary material for: A lexicon obtained and validated by a data-driven approach for organic residues valorization in emerging and developing countries
Source: Front Artif Intell. 2025 Sep 2;8:1557137. doi: 10.3389/frai.2025.1557137 (PMC12436369; doi:10.3389/frai.2025.1557137)
Supplement: Supplementary file 1 [file Data_Sheet_1.docx]

**Appendix**

**Web of science Core collection query: WOS, FSTA and Biosis**

TS = (“sewage sludge” OR "crop residue*" OR "agricultural waste" OR "industrial waste" OR "food waste" OR "household waste" OR "organic waste" OR "urban waste" OR "co-product*" OR "by-product*" OR "biomass" OR "organic waste product*" OR mulch OR digestate* OR compost*) AND TS = (decomposition OR fermentation OR anaerobic OR aerobic OR methanisation OR composting OR vermicomposting OR fertilization OR bokashi OR biodegradation OR mineralization OR recycling OR "agricultural valuation" OR biotransformation OR mulching) AND TS = (africa OR "acp countries" OR "central america" OR "south america" OR "latin america" OR "south east asia" OR "south asia" OR afghanistan OR angola OR albania OR argentina OR armenia OR antigua OR azerbaijan OR burundi OR benin OR "burkina faso" OR bangladesh OR bosnia OR belarus OR belize OR bolivia OR brazil OR bhutan OR botswana OR "central african republic" OR china OR "ivory coast" OR cameroon OR congo OR colombia OR comoros OR "cape verde" OR "costa rica" OR cuba OR djibouti OR dominica OR "dominican republic" OR algeria OR ecuador OR egypt OR eritrea OR ethiopia OR fiji OR micronesia OR gabon OR georgia OR ghana OR guinea OR gambia OR grenada OR guatemala OR guyana OR honduras OR haiti OR indonesia OR india OR iran OR iraq OR jamaica OR jordan OR kazakhstan OR kenya OR kyrgyzstan OR cambodia OR kiribati OR "lao people's democratic republic" OR lebanon OR liberia OR libya OR "saint lucia" OR "sri lanka" OR lesotho OR morocco OR moldova OR madagascar OR maldives OR mexico OR "marshall islands" OR "north macedonia" OR mali OR myanmar OR montenegro OR mongolia OR mozambique OR mauritania OR montserrat OR mauritius OR malawi OR malaysia OR namibia OR niger OR nigeria OR nicaragua OR niue OR nepal OR nauru OR pakistan OR panama OR peru OR philippines OR palau OR "papua new guinea" OR " Democratic People's Republic of Korea" OR "north korea" OR paraguay OR "palestinian territory" OR rwanda OR sudan OR senegal OR "saint helena, ascension and tristan da cunha" OR "solomon islands" OR "sierra leone" OR “el Salvador” OR somalia OR serbia OR "south sudan" OR "sao tome and principe" OR suriname OR eswatini OR "syrian arab republic" OR chad OR togo OR thailand OR tajikistan OR tokelau OR turkmenistan OR "timor-leste" OR tonga OR tunisia OR turkey OR tuvalu OR tanzania OR uganda OR ukraine OR uzbekistan OR "saint vincent and the grenadines" OR venezuela OR "vietnam" OR vanuatu OR "wallis and futuna" OR samoa OR yemen OR "south africa" OR zambia OR zimbabwe).

**Agritrop**

(boue* OU "résidu* de culture*" OU "déchet* agricole" OU "déchet* industriel" OU "déchet* alimentaire" OU "déchet* ménager" OU "déchet* organique" OU "déchet* urbain" OU coproduit* OU "sous-produit*" OU biomasse OU PRO* OU paillis OU digestat* OU compost) ET (décomposition OU fermentation OU anaérobie OU aérobie OU méthanisation OU compostage OU vermicompostage OU fertilisation OU bokashi OU biodégradation OU minéralisation OU recyclage OU “valorisation agricole”OU biotransformation OU paillage) ET (afrique OU "pays acp" OU "amérique centrale" OU "amérique du sud" OU "amérique latine" OU "asie du sud-est" OU "asie du sud" OU afghanistan OU angola OU albanie OU argentine OU arménie OU antigua OU azerbaïdjan OU burundi OU bénin OU "burkina faso" OU bangladesh OU bosnie OU biélorussie OU belize OU bolivie OU brésil OU bhoutan OU botswana OU "république centrafricaine" OU chine OU "côte d'ivoire" OU cameroun OU congo OU colombie OU comores OU "cap-vert" OU "costa rica" OU cuba OU djibouti OU dominique OU "république dominicaine" OU algérie OU équateur OU égypte OU érythrée OU ethiopie OU fidji OU micronésie OU gabon OU géorgie OU ghana OU guinée OU gambie OU grenade OU guatemala OU guyane OU honduras OU haïti OU indonésie OU inde OU iran OU irak OU jamaïque OU jordanie OU kazakhstan OU kenya OU kirghizistan OU cambodge OU kiribati OU « république démocratique populaire lao » OU liban OU libéria OU libye OU “sainte lucie” OU “sri lanka” OU lesotho OU maroc OU moldavie OU madagascar OU maldives OU mexique OU "îles marshall" OU "macédoine du nord" OU mali OU myanmar OU monténégro OU mongolie OU mozambique OU mauritanie OU montserrat OU maurice OU malawi OU malaisie OU namibie OU niger OU nigeria OU nicaragua OU niue OU népal OU nauru OU pakistan OU panama OU pérou OU philippines OU palau OU “papouasie-nouvelle-guinée” OU “corée, république populaire démocratique” OU “corée du nord” OU paraguay OU “territoire palestinien” OU rwanda OU soudan OU sénégal OU “sainte Hélène, ascension et tristan da cunha” OU "îles salomon" OU "sierra leone" OU el salvador OU somalie OU serbie OU "soudan du sud" OU "sao tomé et principe" OU suriname OU eswatini OU "république arabe syrienne" OU tchad OU togo OU thaïlande OU tadjikistan OU tokelau OU turkmenistan OU "timor-leste" OU tonga OU tunisie OU turquie OU tuvalu OU tanzanie OU ouganda OU ukraine OU ouzbekistan OU "saint vincent et les grenadines" OU venezuela OU "viet nam" OU vanuatu OU "wallis et futuna" OU samoa OU yemen OU "afrique du sud" OU zambie OU zimbabwe)

**Scopus:**

Due to a very high number obtained with the equivalent of WoS query, the following query was used for scopus with subject area=environmental sciences or agricultural. Then, only articles, reviews and conference paper were selected.

TITLE-ABS-KEY ( "sewage sludge" OR "crop residue*" OR "agricultural waste" OR "industrial waste" OR "food waste" OR "household waste" OR "organic waste" OR "urban waste" OR "co-product*" OR "by-product*" OR "biomass" OR "organic waste product*" OR mulch OR digestate* OR compost* ) AND TITLE-ABS-KEY ( decomposition OR fermentation OR anaerobic OR aerobic OR methanisation OR composting OR vermicomposting OR fertilisation OR bokashi OR biodegradation OR mineralisation OR recycling OR "agricultural valuation" OR biotransformation OR mulching ) AND ( LIMIT-TO ( SUBJAREA , "ENVI" ) OR LIMIT-TO ( SUBJAREA , "AGRI" ) ) AND ( LIMIT-TO ( DOCTYPE , "ar" ) OR LIMIT-TO ( DOCTYPE , "re" ) OR LIMIT-TO ( DOCTYPE , "cp" ) )

**Google scholar, HAL, Cairn.info, AGRIS:**

Advanced research was not available on free databases; the research was thus conducted with a general query on the topic which was:

- In French : « biotransformation et valorisation en agriculture dans les contextes des pays du Sud »
- In English « biotransformation et valorization in agriculture in low-income countries» in English. The query was then tested by adding Africa, Latin America, then South-East Asia ».
